# Poly-LacNAc as an Age-Specific Ligand for Rotavirus P[11] in Neonates and Infants

**DOI:** 10.1371/journal.pone.0078113

**Published:** 2013-11-11

**Authors:** Yang Liu, Pengwei Huang, Baoming Jiang, Ming Tan, Ardythe L. Morrow, Xi Jiang

**Affiliations:** 1 Division of Infectious Diseases, Cincinnati Children's Hospital Medical Center, Cincinnati, Ohio, United States of America; 2 Division of Biostatistics and Epidemiology, Cincinnati Children's Hospital Medical Center, Cincinnati, Ohio, United States of America; 3 Perinatal Institute, Cincinnati Children's Hospital Medical Center, Cincinnati, Ohio, United States of America; 4 Department of Pediatrics, University of Cincinnati College of Medicine, Cincinnati, Ohio, United States of America; 5 Gastroenteritis and Respiratory Viruses Laboratory Branch, Centers for Disease Control and Prevention, Atlanta, Georgia, United States of America; University of Pittsburgh, United States of America

## Abstract

Rotavirus (RV) P[11] is an unique genotype that infects neonates. The mechanism of such age-specific host restriction remains unknown. In this study, we explored host mucosal glycans as a potential age-specific factor for attachment of P[11] RVs. Using *in vitro* binding assays, we demonstrated that VP8* of a P[11] RV (N155) could bind saliva of infants (60.3%, N = 151) but not of adults (0%, N = 48), with a significantly negative correlation between binding of VP8* and ages of infants (*P*<0.01). Recognition to the infant saliva did not correlate with the ABO, secretor and Lewis histo-blood group antigens (HBGAs) but with the binding of the lectin *Lycopersicon esculentum* (LEA) that is known to recognize the oligomers of N-acetyllactosamine (LacNAc), a precursor of human HBGAs. Direct evidence of LacNAc involvement in P[11] binding was obtained from specific binding of VP8* with homopolymers of LacNAc in variable lengths through a glycan array analysis of 611 glycans. These results were confirmed by strong binding of VP8* to the Lec2 cell line that expresses LacNAc oligomers but not to the Lec8 cell line lacking the LacNAc. In addition, N155 VP8* and authentic P[11] RVs (human 116E and bovine B223) hemagglutinated human red blood cells that are known to express poly-LacNAc. The potential role of poly-LacNAc in host attachment and infection of RVs has been obtained by abrogation of 116E replication by the PAA-conjugated poly-LacNAc, human milk, and LEA positive infant saliva. Overall, our results suggested that the poly-LacNAc could serve as an age-specific receptor for P[11] RVs and well explained the epidemiology that P[11] RVs mainly infect neonates and young children.

## Introduction

Attachment to a host receptor is the essential first step for many bacterial and viral pathogens to initiate an infection [1]. As carbohydrates, the histo-blood group antigens (HBGAs), including those in the ABO, secretor and Lewis families, distribute abundantly on the surfaces of red blood cells and mucosal epithelia, serving as receptors of some pathogens [2]. These HBGAs are also present in soluble form in biologic fluids such as saliva and milk [3]. HBGAs are synthesized by glycosyltransferases from a series of precursor structures by stepwise addition of monosaccharide units [4]. Understanding human HBGAs as receptors of pathogens has been significantly advanced over the past decade through study of human noroviruses, a major cause of acute gastroenteritis. Human noroviruses are diverse in recognizing variable HBGA [5]–[8]. Direct evidence regarding HBGAs in host susceptibility/resistance to norovirus infection has been obtained by human volunteer challenge studies and outbreak investigations [9], [10].

Recently, human rotaviruses (RVs), another major cause of severe diarrhea in children, have also been found to recognize the human HBGAs as potential receptors or attachment factors similar to that of human noroviruses. The RV-HBGA interaction was first demonstrated by binding of recombinant spike VP8* proteins of major human RVs to specific HBGAs [11]. Strains in the P[4] and P[8] genotypes share the common antigens of Lewis b (Le^b^) and H type 1, while strains of the P[6] genotype bind the H type 1 antigen only. The bindings were demonstrated using human saliva, milk, and synthetic HBGA oligosaccharides and the binding was also observed when authentic viruses from a cell culture derived P[8] (Wa) RV were tested.

Following the first study, a phylogenetic analysis of RVs based on the VP8* sequences was performed. These studies demonstrated that RVs can be grouped into five P genogroups (P[I]–P[V]) with clear segregation between human and animal RVs in which P[I] and P[V] mainly infect animals, P[II] infects humans, while P[III] and P[IV] infect both animals and humans [12]. The three major P genotypes of human RVs (P[4], [6] and [8]) recognizing the H-related antigens (Le^b^ and H type 1) described in the first study were all clustered in P[II], suggesting HBGAs as an important determinant of host ranges of RVs. Another study on three genotypes (P[9], [14] and [25]) in P [III] resulted in discovery of a new HBGA binding pattern of RVs; all the three genotypes recognized the type A antigen distinct from the P[II] RVs [12]. In addition, the VP8* of the three P[III] RVs bound the A antigens of the porcine and bovine mucins, suggesting the A antigen as a shared ligand for cross-species transmission of RVs. Direct evidence of the RV-HBGA interaction came from an x-ray crystallography of VP8* of a P[14] RV in complex with the A-oligosaccharides showing the HBGA-binding site of RV. Further study demonstrated a specific inhibition of RV infection in cell culture by an anti-A monoclonal antibody, suggesting the A antigen as a receptor of the P[14] RV [13].

The recognition of carbohydrate receptors by RVs can be traced back to the finding of the sialic acid as a receptor for some animal RVs [14], [15], suggesting that the recognition of a carbohydrate receptor could be a common feature of RVs. It also was found that the same receptor binding interfaces of the A-binding human RVs (P[14]) are shared with the sialic acid-depended animal RVs in the VP8* proteins [13], suggesting a convergent structure for carbohydrate interactions in evolution of RVs. RVs are highly adaptive viruses that infect humans and different animals. While animal RVs may recognize animal specific carbohydrates, human RVs recognize human HBGAs. The adaptive nature of RVs and the polymorphic human HBGAs prompted us to look for other potential binding patterns of RVs.

In this study we demonstrated the carbohydrate binding pattern of another genotype, P[11] of human RVs. P[11] is the sole genotype in P[IV] genogroup that mainly infects cattle and humans with a preference of neonates. The genetic segregation of P[11] (P[IV]) from the other RV genotypes and genogroups and its unique host range for neonates prompted us to search for a potential age specific ligand for P[11] RVs. By *in vitro* binding assays of RV surface spike protein VP8* with large numbers of saliva samples from neonates/infants and adults as well as a large carbohydrate array, we have demonstrated that P[11] RVs specifically recognize a precursor of human HBGAs, the poly-LacNAc, that are available in mucosa of neonates and infants as potential receptors for P[11] RVs. The observed binding specificity and its biologic relevance was further proved by cell culture based binding and infection assays.

## Materials and Methods

### Protein expression and purification

The VP8* sequences (amino acids 46-231) of a human RV P[11] strain N155 (GenBank accession: EU200796) were synthesized by GenScrip (GenScript USA Inc., Piscataway, NJ), and then sub-cloned into the expression vector PGEX-4T-1(GE Healthcare Life Sciences, Piscataway, NJ) between the BamH I and Sal I sites. After confirmation of the inserts through DNA sequencing, the GST-VP8* fusion protein was expressed in *E. coli* BL21 with induction of IPTG (0.4 mM) at room temperature (∼22°C) overnight. The GST-VP8* fusion protein was purified using Glutathione Sepharose 4 Fast Flow (GE Healthcare Life Sciences, Piscataway, NJ) according to the manufacturer's protocol. Briefly, bacterial lysates were mixed with the sepharose beads for 2 h at room temperature to allow the GST fused VP8* proteins binding to the beads. After washing the beads with PBS for 4–6 times, the GST fusion VP8* proteins were eluted by glutathione elution buffer (10 mM reduced glutathione, 50 mM Tris-Cl, PH 8.0).

### Saliva collection

Written informed consent was obtained from the parents of the infants. Samples were collected from infants born in Cincinnati, Ohio under IRB-approved protocols (Cincinnati Children's Hospital Medical Center Institutional Review Board study# 2008-0463 and 2008-1131). Whole saliva was collected from preterm infants <33 weeks of age at day 8 of life who were enrolled in two hospital neonatal intensive care units (Novel Biomarkers Cohort). Collection was conducted early in the morning by trained clinical nurses. Two sterile cotton swabs were placed between the check and the gum and held in place until saturated. A similar procedure was used for collection of whole saliva from healthy, term infants enrolled in a longitudinal study of breastfeeding and infant health (GEHM Study, HD13021), at standardized postnatal intervals (weeks of life 2, 4, 13, 26, and 52). For the latter study, samples were collected by two trained research staff between 9 am and noon. Immediately after collection, samples were refrigerated, and then placed in cryogenic freezers in barcoded vials until tested. Infants from both studies were predominantly white, with approximately one-quarter African-American infants.

### Saliva-based binding assays

Saliva samples from adults and infants were tested for potential age-specific ligand in neonates for P[11] RVs by in vitro binding assays . Forty-eight saliva from adults, representing different ABO, secretor and Lewis types described in our previous studies, were included [5], [16]. Seventy-two saliva samples from neonates at day 8 after birth and 79 saliva samples from infants at weeks 4, 13, 26, and 52 after birth were studied. Saliva samples were boiled for 10 min to inactivate potential antibodies against RVs that could interfere with the binding assays. The samples then were diluted 1∶1,000 and coated on 96-well microtiter plates at 4°C overnight. After blocking with 5% nonfat cow milk, 100 µl of GST-VP8* proteins (10 µg/ml) were added and incubated at 37°C for 1 h. The bound GST-VP8* proteins were detected using a rabbit anti-GST serum at 1∶4000 dilution followed by horseradish peroxidase (HRP)-conjugated goat anti-rabbit IgG (ICN, Aurora, OH). The signal intensities were displayed using the TMB kit according to manufacturer (BD Biosciences, San Diego, CA). In each step, the plates were incubated for 1 h at 37°C and washed five times with PBS-Tween 20 using a plate washer. The HBGA types of each saliva sample were determined by binding assay using monoclonal antibodies specific to H-1, A, B, Le^a^, Le^b^, Le^x^ and Le^y^ antigens as previously described [5]. The saliva samples were also tested for binding by lectin *Lycopersicon esculentum* (LEA) that recognize poly-LacNAc. The biotin-labeled LEA (Sigma-Aldrich Co., St. Louis, MO) in dilution of 1∶1000 was used, which was detected by HRP-conjugated-streptavidin (Jackson ImmunoResearch Laboratories Inc., West Grove, PA) and displayed using the TMB kit (Kierkegaard and Perry Laboratory, Gaithersburg, MD).

### Glycan array screening

The glycan array analysis was performed by the Protein-Glycan Interaction Core at the Emory University under the Consortium for Functional Glycomics. A printed glycan array (version 5.0) consists of 611 glycans in replicates of 6 was used to search for potential carbohydrate ligands for P[11] RVs (http://www.functionalglycomics.org/). The recombinant GST-VP8* proteins were applied to individual glycan arrays at two protein concentrations (20 and 200 µg/ml respectively), and the bound GST-VP8* proteins were detected by using a fluorescent-labeled anti-GST monoclonal antibody. Two sets of relative fluorescent units (RFU) of each glycan at the two concentration were calculated and used to rank the specificity of individual glycans (Table 1) for their interaction with P[11] VP8*.

**Table 1:** Data of top 15 glycans ranked highest in binding to P[11] VP8* among 611 glycans tested in the array analysis.

			200 µg/ml		20 µg/ml	
Glycan#	Glycan Structure	AVG.RANK^b^	Avg.RFU^a^	RANK	Avg.RFU	RANK
589	Galβ1-4GlcNAcβ1-3Galβ1-4GlcNAcβ1-3Galβ1-4GlcNAcβ1-3Galβ1-4GlcNAcβ1-3Galβ1-4GlcNAcβ1-6(Galβ1-4GlcNAcβ1-3Galβ1-4GlcNAcβ1-3Galβ1-4GlcNAcβ1-3Galβ1-4GlcNAcβ1-3Galβ1-4GlcNAβ1-2)Mana1-6(Galβ1-4GlcNAcβ1-3Galβ1-4GlcNAcβ1-3Galβ1-4GlcNAcβ1-3Galβ1-4GlcNAcβ1-3Galβ1-4GlcNAcβ1-2Mana1-3)Manβ1-4GlcNAcβ1-4(Fuca1-6)GlcNAcβ-Sp24	78	13662	87	4468	69
582	Galβ1-4GlcNAcβ1-3Galβ1-4GlcNAcβ1-3Galβ1-4GlcNAcβ1-3Galβ1-4GlcNAcβ1-3Galβ1-4GlcNAcβ1-2Mana1-6(Galβ1-4GlcNAcβ1-3Galβ1-4GlcNAcβ1-3Galβ1-4GlcNAcβ1-3Galβ1-4GlcNAcβ1-3Galβ1-4GlcNAcβ1-2Mana1-3)Manβ1-4GlcNAcβ1-4(Fuca1-6)GlcNAcβ-Sp19	76	14037	89	4069	63
569	Galβ1-4GlcNAcβ1-3Galβ1-4GlcNAcβ1-3Galβ1-4GlcNAcβ1-3Galβ1-4GlcNAcβ1-3Galβ1-4GlcNAcβ1-3Galβ1-4GlcNAcβ1-2Mana1-6(Galβ1-4GlcNAcβ1-3Galβ1-4GlcNAcβ1-3Galβ1-4GlcNAcβ1-3Galβ1-4GlcNAcβ1-3Galβ1-4GlcNAcβ1-3Galβ1-4GlcNAcβ1-2Mana1-3)Manβ1-4GlcNAcβ1-4GlcNAcβ-Sp25	71	6641	42	6438	100
549	Galβ1-4GlcNAcβ1-3Galβ1-4GlcNAcβ1-3Galβ1-4GlcNAcβ1-2Mana1-6(Galβ1-4GlcNAcβ1-3Galβ1-4GlcNAcβ1-3Galβ1-4GlcNAcβ1-2Mana1-3)Manβ1-4GlcNAcβ1-4GlcNAcβ-Sp24	69	13066	83	3609	56
566	Galβ1-4GlcNAcβ1-3Galβ1-4GlcNAcβ1-3Galβ1-4GlcNAcβ1-3Galβ1-4GlcNAcβ1-3Galβ1-4GlcNAcβ1-2Mana1-6(Galβ1-4GlcNAcβ1-3Galβ1-4GlcNAcβ1-3Galβ1-4GlcNAcβ1-3Galβ1-4GlcNAcβ1-3Galβ1-4GlcNAcβ1-2Mana1-3)Manβ1-4GlcNAcβ1-4GlcNAcβ-Sp25	69	15793	100	2385	37
581	GlcNAcβ1-3Galβ1-4GlcNAcβ1-3Galβ1-4GlcNAcβ1-3Galβ1-4GlcNAcβ1-3Galβ1-4GlcNAcβ1-2Mana1-6(GlcNAcβ1-3Galβ1-4GlcNAcβ1-3Galβ1-4GlcNAcβ1-3Galβ1-4GlcNAcβ1-3Galβ1-4GlcNAcβ1-2Mana1-3)Manβ1-4GlcNAcβ1-4(Fuca1-6)GlcNAcβ-Sp19	67	15601	99	2230	35
565	GlcNAcβ1-3Galβ1-4GlcNAcβ1-3Galβ1-4GlcNAcβ1-3Galβ1-4GlcNAcβ1-3Galβ1-4GlcNAcβ1-2Mana1-6(GlcNAcβ1-3Galβ1-4GlcNAcβ1-3Galβ1-4GlcNAcβ1-3Galβ1-4GlcNAcβ1-3Galβ1-4GlcNAcβ1-2Mana1-3)Manβ1-4GlcNAcβ1-4GlcNAcβ-Sp25	66	13077	83	3218	50
580	Galβ1-4GlcNAcβ1-3Galβ1-4GlcNAcβ1-3Galβ1-4GlcNAcβ1-3Galβ1-4GlcNAcβ1-2Mana1-6(Galβ1-4GlcNAcβ1-3Galβ1-4GlcNAcβ1-3Galβ1-4GlcNAcβ1-3Galβ1-4GlcNAcβ1-2Mana1-3)Manβ1-4GlcNAcβ1-4(Fuca1-6)GlcNAcβ-Sp24	64	13458	85	2731	42
551	Galβ1-4GlcNAcβ1-3Galβ1-4GlcNAcβ1-3Galβ1-4GlcNAcβ1-3Galβ1-4GlcNAcβ1-2Mana1-6(Galβ1-4GlcNAcβ1-3Galβ1-4GlcNAcβ1-3Galβ1-4GlcNAcβ1-3Galβ1-4GlcNAcβ1-2Mana1-3)Manβ1-4GlcNAcβ1-4GlcNAcβ-Sp25	64	12757	81	3013	47
587	Galβ1-4GlcNAcβ1-3Galβ1-4GlcNAcβ1-3Galβ1-4GlcNAcβ1-3Galβ1-4GlcNAcβ1-6(Galβ1-4GlcNAcβ1-3Galβ1-4GlcNAcβ1-3Galβ1-4GlcNAcβ1-3Galβ1-4GlcNAβ1-2)Mana1-6(Galβ1-4GlcNAcβ1-3Galβ1-4GlcNAcβ1-3Galβ1-4GlcNAcβ1-3Galβ1-4GlcNAcβ1-2Mana1-3)Manβ1-4GlcNAcβ1-4(Fuca1-6)GlcNAcβ-Sp24	59	11561	73	2937	46
588	GlcNAcβ1-3Galβ1-4GlcNAcβ1-3Galβ1-4GlcNAcβ1-3Galβ1-4GlcNAcβ1-3Galβ1-4GlcNAcβ1-6(GlcNAcβ1-3Galβ1-4GlcNAcβ1-3Galβ1-4GlcNAcβ1-3Galβ1-4GlcNAcβ1-3Galβ1-4GlcNAβ1-2)Mana1-6(GlcNAcβ1-3Galβ1-4GlcNAcβ1-3Galβ1-4GlcNAcβ1-3Galβ1-4GlcNAcβ1-3Galβ1-4GlcNAcβ1-2Mana1-3)Manβ1-4GlcNAcβ1-4(Fuca1-6)GlcNAcβ-Sp24	56	8415	53	3755	58
545	Fuca1-2Galβ1-4GlcNAcβ1-3Galβ1-4GlcNAcβ1-2Mana1-6(Fuca1-2Galβ1-4GlcNAcβ1-3Galβ1-4GlcNAcβ1-2Mana1-3)Manβ1-4GlcNAcβ1-4GlcNAcβ-Sp24	51	12905	82	1368	21
579	GlcNAcβ1-3Galβ1-4GlcNAcβ1-3Galβ1-4GlcNAcβ1-3Galβ1-4GlcNAcβ1-2Mana1-6(GlcNAcβ1-3Galβ1-4GlcNAcβ1-3Galβ1-4GlcNAcβ1-3Galβ1-4GlcNAcβ1-2Mana1-3)Manβ1-4GlcNAcβ1-4(Fuca1-6)GlcNAcβ-Sp24	49	11259	71	1667	26
75	Fuca1-2Galβ1-4GlcNAcβ1-3Galβ1-4GlcNAcβ1-3Galβ1-4GlcNAcβ-Sp0	46	10882	69	1504	23
547	GlcNAcβ1-3Galβ1-4GlcNAcβ1-3Galβ1-4GlcNAcβ1-2Mana1-6(GlcNAcβ1-3Galβ1-4GlcNAcβ1-3Galβ1-4GlcNAcβ1-2Mana1-3)Manβ1-4GlcNAcβ1-4GlcNAcβ-Sp25	46	9669	61	1955	30

a The highest and lowest points from each set of six replicates were removed to eliminate potential false hits. Binding specificity is shown in mean relative fluorescence units (RFU) of binding to the four replicates of each glycan printed on the array.

b The fluorescent signals from each glycan were then normalized to the maximum signal from each assay, and the glycans were ordered according to the average ranking for the two assays (20 and 200 µg/ml).

Spacer Arm abbreviations: Sp0: -CH2CH2NH2; Sp19: EN or NK; Sp24: KVANKT; Sp25: VANK.

### Oligosaccharide-based binding assay

Synthetic polyacrylamide polymer (PAA) conjugated oligomers of LacNAc units with variable lengths or fucosyl modifications (Table 2) were used to study their specificity as a ligand for P[11] RVs. Briefly, microtiter plates (Dynex Immulon; Dynatech, Franklin, MA) were coated with recombinant VP8* proteins (10 µg/ml) at 4°C overnight. After blocking with 5% nonfat cow milk, synthetic poly-LacNAc (PAA)-biotin conjugates (Table 2) obtained from the Consortium for Functional Glycomics or from GlycoTech Inc. (Gaithersburg, MD) were added at serial dilutions and incubated at 4°C overnight. The bound oligosaccharides were then detected using HRP-conjugated-streptavidin (Jackson ImmunoResearch Laboratories Inc., West Grove, PA) and displayed using the TMB kit (Kierkegaard and Perry Laboratory, Gaithersburg, MD).

**Table 2:** Oligosaccharides used in ELISA for binding to P[11] VP8*.

Common name	Oligosaccharide structure
LacNAc-PAA	Galβ1-4GlcNAcβ-PAA
DiLN-PAA	(Galβ1-4GlcNAcβ1-3)_2_β-PAA
TriLN-PAA	(Galβ1-4GlcNAcβ1-3)_3_β-PAA
2′F-Di-LN-PAA	Fucα1-2 (Galβ1-4GlcNAcβ1-3)_2_β-PAA
3′GN-LN-PAA	GlcNAcβ1-3 Galβ1-4GlcNAcβ-PAA
Lex-PAA	Galβ1-4(Fucα1-3)GlcNAcβ-PAA
Tri-Lex-PAA	(Galβ1-4(Fucα1-3)GlcNAcβ1-3)_3_β-PAA

### Binding assay of VP8* to CHO cell mutants

Two mutant cell lines (Lec2 and Lec8) of the Chinese hamster ovary (CHO) cells were studied for their presence (Lec2) or absence (Lec8) of poly-LacNAc on their surfaces [17]. The Lec2 and Lec8 mutant cells that were kindly provided by Dr. Pamela Stanley were grown in monolayer in α-MEM medium supplemented with ribonucleosides, deoxyribonucleosides (Gibco), 10% FBS, 100 unit/mL penicillin, 100 mg/mL streptomycin, and maintained at 37°C with 5% CO_2_. The binding of VP8* to the cells was detected by immunofluorescent method. Briefly, the monolayer Lec2 and Lec8 cells were grown on poly-L-lysine treated coverslips to a 90% confluence, and then fixed with 4% paraformaldehyde. After blocking with 3% BSA, GST-VP8* fusion protein (100 µg/mL) was added. The binding signal of VP8* on the cell surface was detected using a rabbit anti-GST antibody followed by the FITC-conjugated goat anti-rabbit second antibody.

### Virus propagation, inhibition and infectivity assay

Two P[11] RVs, a human strain 116E and a bovine strain B223 [18], were studied for inhibition of replication in cell cultures by the oligosaccharide of PAA- TriLN and by human milk and saliva using procedures described previously [19]. Confluent MA-104 cell monolayers were grown on coverslips in 24-well plates. After rinse twice with serum-free DMEM, 500 µl of serum-free DMEM was added to each well and the plates were incubated at 37°C for 3 h. For blocking of infection, 300 fluorescent forming units (FFU)/10 µl of virus were treated with equal volume of serial dilutions of human milk, LEA positive neonate saliva or PAA-TriLN at room temperature for 30 min. An adult and a LEA negative neonate saliva which did not show bindings with P[11] VP8* were used as controls. Following chilling of all reagents and the 24-well plates on ice, duplicated wells were inoculated with the virus-oligosaccharide or virus-saliva/milk mixtures on ice with continuous rocker platform agitation for 1 h. The inoculum was then removed and the cells were washed twice with ice-cold serum-free DMEM. After replacement with 500 µl serum-free DMEM, the plates were warmed to 37°C and incubated in a 5% CO_2_ incubator for 18 to 20 h prior to quantification of infected cells by immunofluorescence with a rabbit anti-rotavirus antibody followed by a FITC-labelled goat anti-rabbit secondary antibody.

### Hemagglutination assay

Human red blood cells (RBC) of A_1_, A_2_, B and O type (Immucor, Inc., Norcross, GA) were washed twice in PBS before being tested for hemagglutination with VP8* or authentic RVs. An equal volume of 50 µL of 1% RBC were added to 50 µL of two-fold serial dilutions of either the GST-VP8* fusion proteins or virus on 96-well V-bottom plates. Agglutination was determined after 2 h of incubation at 4°C.

### Statistical analysis

The correlation analysis was conducted using the Pearson analysis, and the binding signal difference of VP8* to neonate saliva between 2–13 weeks and 26–52 weeks was assessed by a two-tailed t test. P values of <0.05 were considered significant.

## Results

### VP8* of N155 (P[11] RV) binds saliva exclusively from neonates/infants but not from adults

**Figure 1:**
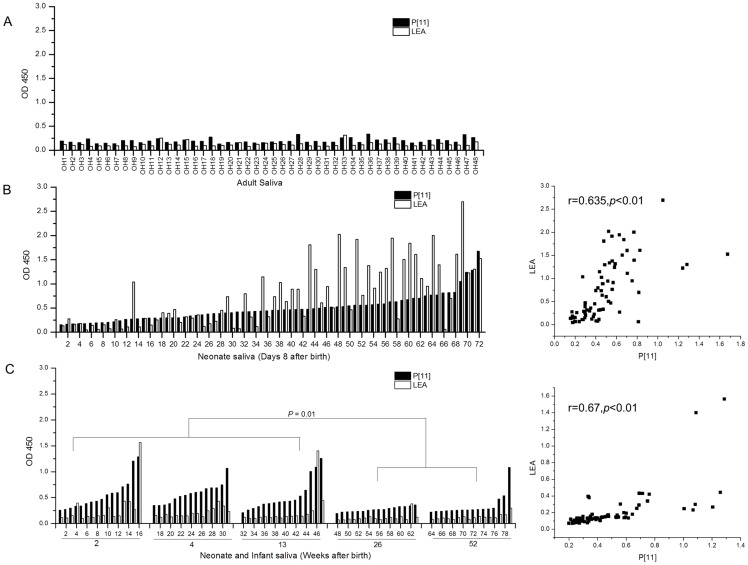
Binding of recombinant N155 RV VP8* protein to saliva samples collected from adults (A), neonates (B) and infants (C). The neonate saliva samples were collected at day 8 after birth and the infant saliva samples were collected at weeks 2, 4, 13, 26 and 52 after birth respectively. The saliva samples also were tested for binding by lectin *Lycopersicon esculentum* (LEA) that recognizes poly-LacNAc. None of the 48 adult saliva revealed significant binding with either P[11] VP8* or LEA (OD<0.35) but significant binding signals were observed in saliva of neonates and infants. The binding signals of P[11] VP8* showed a positive correlation with that of LEA among the neonate and infant saliva tested (*P*<0.01). The binding signals of P[11] VP8* were negatively correlated with the development stages of the infants (*P*<0.01), with binding activities in younger (weeks 2–13) significantly higher than that in older (weeks 26 and 52) infants (*P* = 0.01).

To explore potential age-specific receptors for P[11] RVs, we performed saliva binding assays using recombinant GST-VP8* fusion protein as described previously [11]. The yields of the fusion proteins were 5–10 mg/L of bacteria culture and high purity of the GST-VP8* protein was obtained for the binding assays (data not shown). Only marginal binding signals (OD<0.35) were detected among the 48 well-characterized adult saliva samples (Fig. 1A). However, strong binding signals were observed when the two sets of neonate/infant saliva samples were tested (60.3% ODs>0.35, N = 151). High binding signals showed no correlation with the ABO, secretor and Lewis types (data not shown). The first set of infant saliva were collected earlier in life (day 8 after birth) and a higher rate (68.1%) of the samples showed a binding signal above 0.35 (Fig. 1B). The second set of infant saliva was collected from a wide age range (weeks 2, 4, 13, 26 and 52 after birth) and an average 53.2% of positive binding (OD>0.35) was observed. Moreover, the binding of P[11] VP8* with the saliva showed a significantly negative correlation with the age of infants (*P*<0.01). While most of the positive binding occurred in the early development stages (weeks 2, 4 and 13), only sporadic binding was found in saliva collected from infants at weeks 26 and 52 (Fig. 1C).

### P[11] VP8* binds poly-LacNAc

**Figure 2:**
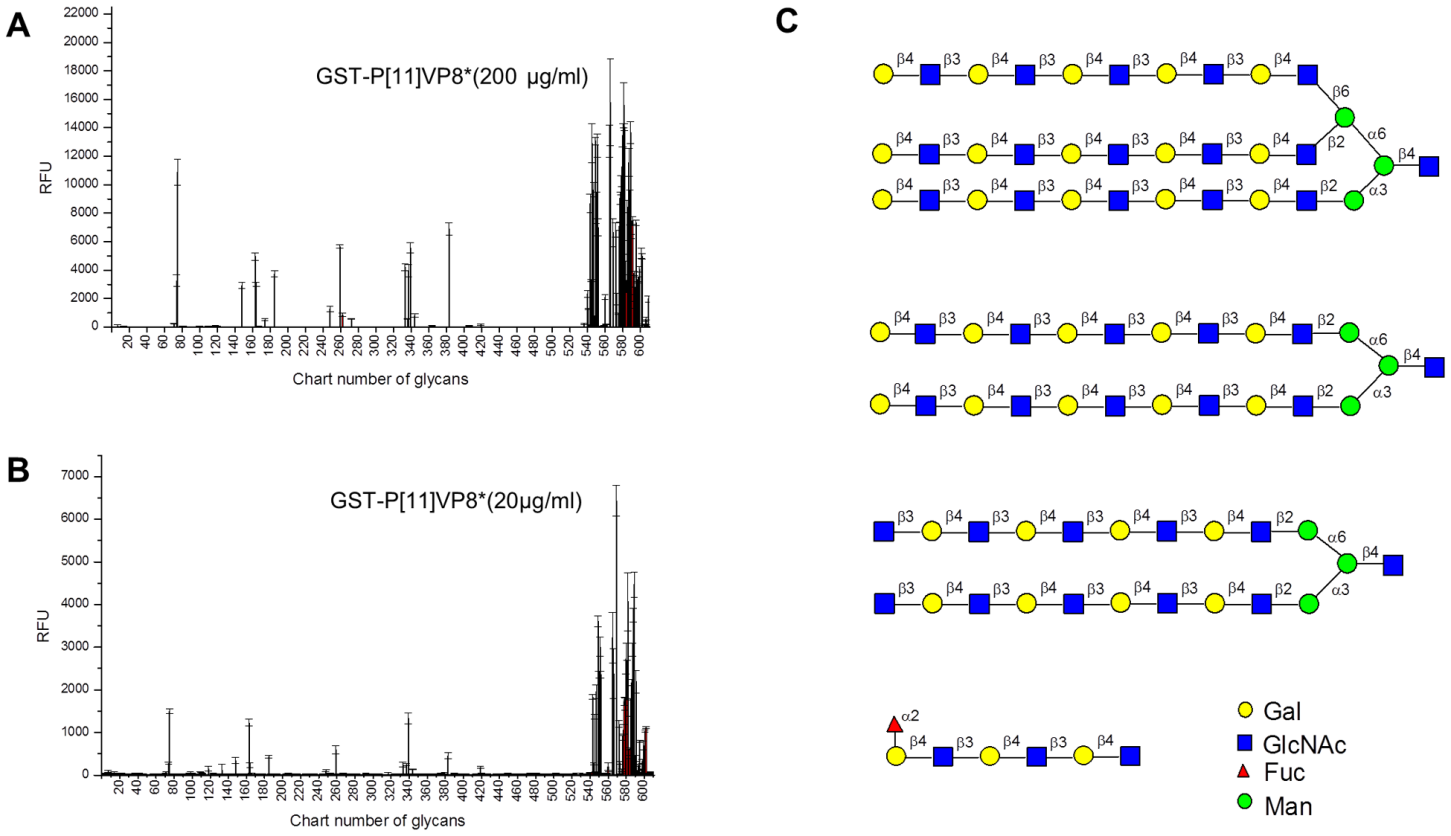
Binding of N155 RV VP8* to oligosaccharide glycans by a glycan array assay. The printed array consists of 611 glycans in replicates of 6. Duplicate sets with the VP8* protein concentrations at 20 (A) and 2 (B) µg/ml were tested. The relative fluorescent units (RFU) corresponded to strength of binding of individual glycans and the error bar represented the standard deviation of the replicates. The identities of the glycans used in the array (version 5.0) are available on line (http://www.functionalglycomics.org/). The oligosaccharide glycans ranked highest in binding to VP8* were listed in the table 1. The schematic structures of representative high ranking glycans are shown (C).

To determine the ligand for P[11] VP8* in the infant saliva, we performed an array assay consisted of 611 oligosaccharide glycans, and similar binding patterns of the P[11] GST-VP8* fusion protein at both 20 and 200 µg/ml were observed (Fig. 2A–B). A group of glycans with a common feature, linear or branched polymers of the LacNAc disaccharide unit in variable lengths, ranked the highest (Table 1). Strong binding always occurred to the poly-LacNAc oligosaccharides without further terminal modifications. The fucose (α1–2) linked to the terminal Gal of poly-LacNAc did not affect the binding (Table 1). LacNAc polymers containing other terminal modifications, such the sialic acid (Neu5Ac) or GalNAc adding to the terminal galactose resulted in reduction of the binding. LacNAc is a precursor of human HBGAs that express abundantly in neonates and young children. Thus, the glycans of LacNAc polymers could be an age-specific ligand available to P[11] RVs before the maturation of HBGAs of neonates.

**Figure 3:**
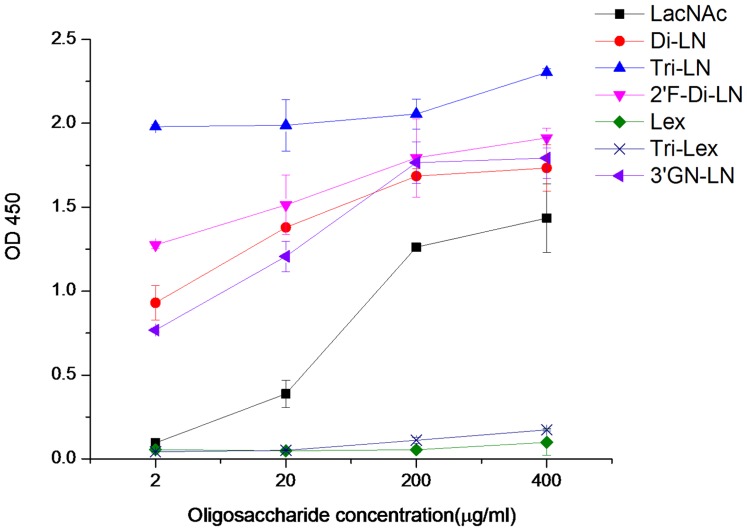
Binding of P[11] VP8* to different forms and modifications of poly-LacNAc by ELISA. LacNAc, a single unit of disaccharide Gal-GlcNAc; Di-LN, two repeats of the Gal-GlcNAc disaccharide units; Tri-LN, three repeats of the Gal-GlcNAc units; 2F-Di-LN, Di-LN with a α1–2 linked fucose at a terminal Gal. 3′GN-LN, a trisaccharide terminated with the GlcNAc residue. Two oligosaccharides, Lex and Tri-Lex, corresponded to the disaccharides of LacNAc and the Tri-LN but with a further fucose modification linked (α1–3) to the GlcNAc residues. These oligosaccharides were conjugated with polyacrylamide (PAA) and were tested with increased concentrations from 2 to 400 µg/ml.

Results of the oligosaccharide-based binding assay exhibited a dose-dependent binding to the LacNAc disaccharides, further confirming that LacNAc is a basic unit recognized by P[11] RVs. It was noted that the binding signals of P[11] VP8* increased significantly with the numbers of LacNAc repeats, in which the three repeats (TriLN-PAA) showed the highest signals followed by the two repeats (DiLN-PAA) and then the single copy (LacNAc-PAA). No significant difference of P[11] binding to LacNAc polymers terminated with either a Gal or a GlcNAc residue was observed. A fucose (α1–2) linked to the terminal Gal did not affect the P[11] VP8* binding but a fucose (α1–3) linked to the GlcNAc of a poly-LacNAc totally abolished the binding (Fig. 3).

### Poly-LacNAc is also responsible for P[11] binding to neonatal saliva

To further study the role of LacNAc in P[11] RV host range, we performed binding assays of the 151neonate/infant saliva samples with lectin *Lycopersicon esculentum* (LEA)that recognizes but is not limited to the poly-LacNAc. Interestingly, none of the 48 adult saliva samples demonstrated any binding (Fig. 1A). However, a strong correlation of binding of P[11] with that of the LEA was observed for the neonate/infant saliva (*P*<0.01, Fig. 1B and C), indicating that it is poly-LacNAc in the neonate/infant saliva responsible for the age-specific binding of P[11]VP8*.

### P[11] VP8* binds poly-LacNAc on the surface of CHO cells

**Figure 4:**
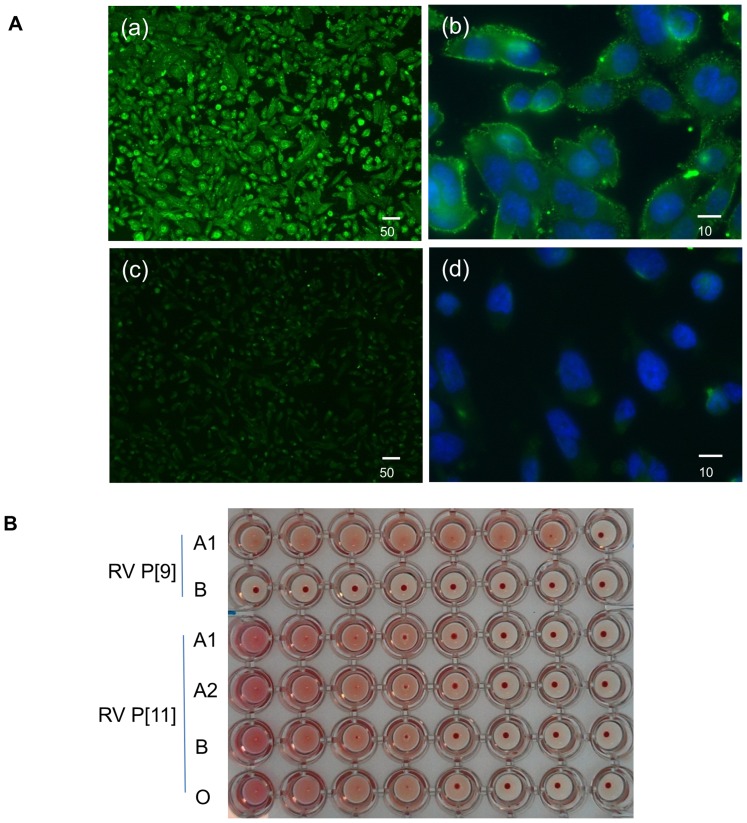
Interactions of P[11]VP8* with LacNAc at cellular level. (A) Binding of GST-VP8* to CHO mutants Lec2 (a)–(b) and Lec8 (c)–(d). CHO mutant Lec2 cells are known to express surface glycans with exclusive end LacNAc oligmeres, while mutant Lec8 cells are absent of LacNAc glycans. Clear binding signals of P[11] VP8* to the Lec2 but not Lec8 cells are observed. (B) Hemagglutination of P[11] RVs with human red blood cells (RBC). Cell culture adapted P[11] RV (B223) revealed agglutination with RBCs of all ABO types. The P[9] RVs which are known to bind type A antigens cause hemagglutination with type A RBCs only.

Two Chinese hamster ovary (CHO) cell lines, the Lec2 and Lec8 cells, were chosen for their well-characterized cell surface glycomics to explore the specific binding for P[11] VP8*. The Lec2 cells have an inactive cytidine monophosphate (CMP)-sialic acid Golgi transporter which resulted in a cell surface complex/hybrid N-glycans mainly consist of poly-LacNAc oligosaccharides on the cell surfaces, while the Lec8 cells do not express LacNAc because of a mutant UDP-Gal Golgi transporter gene [17]. Using immune fluorescence staining methods, the P[11] VP8* displayed specific binding to the Lec2 cells but not to the Lec8 cells (Fig. 4A), indicating that P[11] VP8* was selectively attached to the cell-surface poly-LacNAc of the Lec2 cells.

### Both P[11] VP8* protein and authentic virus hemagglutinated human RBCs

Hemagglutination assays of P[11] VP8* as well as two authentic P[11] RVs (116E and B223) was conducted. A strong agglutination of human RBCs was obtained for both the P[11] VP8* protein and the authentic P[11] RVs, irrespective of the ABO phenotypes, while the VP8* of a P[9] RV, a known type A antigen binder, agglutinated the type A human RBCs only (Fig. 4B). The observed unique hemagglutinating property of P[11] RVs was consistent with the ubiquitous distribution of the poly-LacNAc relevant antigens (group i and I antigens) found on the surface of human erythrocyte cells [20].

### Inhibition of P[11] RV replication by LacNAc polymers, human milk and neonate saliva

**Figure 5:**
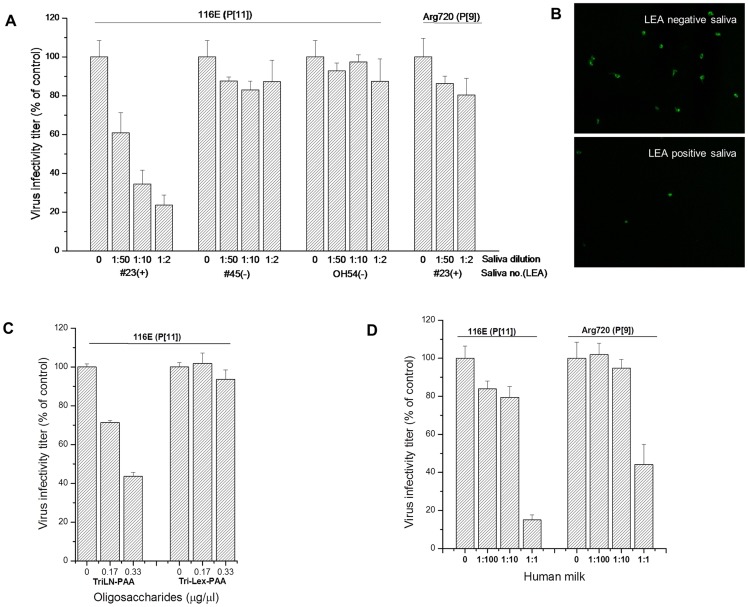
Inhibition of P[11] RV replication in cell cultures by poly-LacNAc conjugates, saliva and human milk. The assay was performed on P[11] RV 116E growing in MA104 cells. Viruses were incubated with poly-LacNAc conjugates, saliva or human milk at room temperature for 30 min before being inoculated on MA104 cells. (A) For blocking by saliva, a LEA positive (#23) and a negative (#45) neonate were tested. A control for the positive neonate saliva (#23) in blocking a type A binder of P[9] RVs was included. (B) Representative immunofluorescence microscopy images of MA104 cells infected with 116E in an untreated control (top) and LEA positive saliva (#23) treated cells (bottom) are shown. (C) The PAA conjugated TriLN with three tandem repeats of LacNAc disaccharide units was used at a final concentration of 0.17 and 0.33 µg/µl respectively. The oligosaccharide Tri-Lex-PAA was used as a control. (D) For blocking by human milk, FPLC fraction F5 of milk 1 which revealed the highest binding signals to P[11] VP8* was used. The inhibition of P[11] RV infectivity was determined by reduction of fluorescent counts between PAA-TriLN, saliva and human milk treated and untreated samples.

To examine the biologic relevance of P[11]VP8* binding to poly-LacNAc, viral replication inhibition assays were performed. The replications of P[11] RV 116E were significantly reduced following incubation of the viruses with a LEA positive neonate saliva (#23) but not with a LEA negative neonate saliva (#45) or with an adult saliva (OH54). A dose-dependent abrogation with up to 76.4% reduction of 116E infectivity by the LEA positive neonate saliva was observed compared to the LEA negative neonate saliva. Moreover the LEA positive saliva (#23) did not inhibit the A-antigen binding RV (Arg720) (Fig. 5A, B). The involvement of poly-LacNAc in RV infection was further demonstrated by a dose-dependent inhibition of 116E incubated with LacNAc polymers (TriLN-PAA) but not with the control oligosaccharide Tri-Lex-PAA (Fig. 5C). Boiled human milk also blocked 116E replication although reductions were also observed for the P[9] Arg720 strain (Fig. 5D).

## Discussion

In this study we demonstrated that the poly-N-acetyllactosamine (poly-LacNAc) could serve as a potential age-specific ligand for attachment and infection of RV P[11] in neonates and infants. A study with similar conclusion was published recently [21]. Evidences of poly-LacNAc as an age-specific ligand in neonates in our study were obtained by the specific binding of P[11] VP8* to saliva of neonates and infants but not of adults and by the significant correlation of binding signals between VP8* and lectin LEA that recognizes poly-LacNAc. The specificity of RV P[11] recognizing poly-LacNAc has been further shown by selective binding of P[11] VP8* to LacNAc polymers by oligosaccharide glycan array and ELISA, by selective binding of P[11] VP8* to surfaces of LacNAc-bearing Lac2 cells but not of LacNAc negative Lac8 cells, and by hemagglutination of both VP8* and authentic P[11] RVs with human RBCs that are known to express LacNAc glycans. The biological relevance of poly-LacNAc recognition by P[11] RVs has been shown by blocking of P[11] RV infection in cell cultures by poly-LacNAc-PAA oligosaccharide (TriLN-PAA) and by human milk and infant saliva that bind P[11] VP8*.

The poly-LacNAc structures occur in mammalian glycoproteins in both N- and O-linked glycans [22]. It is synthesized by repeated alternating additions of N-acetylglucosamine and galactose, catalyzed by beta-1,3-N-acetylglucosaminyltransferases (poly-LacNAc synthase) and beta-1,4-galactosyltransferases, respectively, resulting in multiple repeats of the LacNAc disaccharide unit in each chain [23], [24]. The poly- LacNAc chains represent a backbone for manifold modifications by variable glycotransferases such as the fucosyltransferases, sialyltransferases and sulfotransferases, which further extend to more complex structures [25]–[27], and thus were involved in a variety of biological pathways including immune cell signaling, maintenance of membrane stability, intracellular sorting of proteins, metastasis and development [28].

The roles of poly-LacNAc in metastasis and developmental regulation are most interesting to our study. The carbohydrates on the mucosa of neonates mainly may exist in the form of poly-LacNAc, as demonstrated by the highly occurrence of poly-LacNAc signals in saliva of neonates. However, the abundance and size of the poly-LacNAc chains are often reduced during development [29], [30]. Normally, poly-LacNAc is barely detectable in adult mucosa, except in certain malignant tissues such as colorectal cancer [24], [31]. The ‘disappearance’ of poly-LacNAc in the adult mucosa also could be due to further modification of poly-LacNAc by variable glycotransferases which may affect its interaction with P[11] RVs by epitope-blocking or -masking of the LacNAc residues. Since these glycotransferases are important in maturation of human HBGAs and other glycans, the age of neonates and infants for these genes to turn on could be critical for the different susceptibility of P[11] between neonates/infants and older children/adults. In this study, saliva samples from neonates and infants showed high rates of presence of poly-LacNAc and binding by P[11] VP8* with a negative correlation between P[11] VP8* binding and the development stages of infants. Binding of VP8* to infant saliva collected before weeks 13 showed a significant difference from that of weeks 26 and 52 (*P* = 0.01), indicating an age range to turn off the synthesis of poly-LacNAc or turn on other glycotransferases. Future study to pinpoint these age ranges is of significance. In addition, some individuals remained high binding to P[11] VP8* at week 52 after birth, future study to determine whether the genetic makeup in addition to age variation also contributing to the poly-LacNAc expression is necessary.

We performed binding assay of P[11] VP8* with variable amount of monomeric LacNAc and revealed a typical dose-dependent binding result. We also test poly-LacNAc with either β-galactose or N-acetylglucosamine as the terminal residues and did not observe difference in their binding to P[11] VP8*. Poly-LacNAc with further fucosal modifications either did not affect, such as an addition of α 1,2-fucose to the terminal galactose, or completely abolished the binding, such as the addition of α1, 3-fucose to the N-acetylglucosamine. These results strongly suggest that the disaccharide LacNAc is the basic unit recognized by P[11] RVs. However, in a recent study [21], significantly increased infectivity of P[11] RVs was observed in CHO cells following transfection with the FUT2 gene, which led to a conclusion that the α 1,2-fucose played a major role in P[11] binding and infection. Since there were no binding assay of α 1,2-fucose modified LacNAc were performed in the study, it is difficult to determine whether the fucose was actually added to the LacNAc precursors of the transfected CHO cell surface glycans. Nevertheless, the increased infectivity is not supported by our saliva binding results nor by the epidemiology of P[11] RVs that mainly infect neonates and young children. Future study to clarify this issue may be necessary.

The mechanism of significant higher binding signals of polymers than monomers of LacNAc remains to be determined. First, the increased binding activities of P[11] VP8* to poly-LacNAc suggest increased binding avidities due to multi-repeats in the linear or multi-antennary chains of poly-LacNAc. Alternatively, increased binding signals by LacNAc oligomers could be due to increased freedoms of the terminal LacNAc accessible to VP8* by the increased lengths of the chains. Our findings on the binding properties of P[11] VP8* are similar with that of the Ca2+ independent (S-type) lectins such as the galectin family, which could also bind several LacNAc structures. Likewise, increased binding was reported accompanied with the adding of the LacNAc units on the poly-LacNAc chains [32], [33]. The crystal forms of galectin-1 with biantennary glycans revealed the galectin dimers could be cross-linked by the LacNAc located at the end of the oligosaccharide antenna; however crystals complexed with internal LacNAc units have not yet been reported.

Our study also raises questions regarding the epidemiology of P[11] RVs. For example, although HBGAs related antigens were also found in animal species, the presence and dynamic change of the precursor poly-LacNAc on the gastrointestinal epithelia and their involvement in P[11] infection in cattle remains unknown. A recent study showed that the P[11] RV could also infect sheep [34]. It remains inconclusive whether species-specific antigens and/or age-specific LacNAc are involved in the animal P[11] RV infection in cattle, sheep or other species. Future study with more defined animal mucins is necessary. We also tried to determine the age range for poly-LacNAc after birth of humans, and more follow up studies on better designed infant populations are needed. Finally, study of poly-LacNAc in saliva from infants in the south east Asian countries, particularly in India, is necessary where the P[11] RVs are more prevalent than other areas of the world.
